# Transgene autoexcision in switchgrass pollen mediated by the Bxb1 recombinase

**DOI:** 10.1186/1472-6750-14-79

**Published:** 2014-08-22

**Authors:** Maria N Somleva, Chang Ai Xu, Kieran P Ryan, Roger Thilmony, Oliver Peoples, Kristi D Snell, James Thomson

**Affiliations:** 1 Metabolix, Inc., 21 Erie St., Cambridge, MA 02139, USA; 2 USDA-ARS-CIU, 800 Buchanan St., Albany, CA 94710, USA

**Keywords:** Autoexcision, Bxb1 site-specific recombinase, Developmentally programmed transgene excision, Pollen-mediated gene flow, *Panicum virgatum* L., Switchgrass

## Abstract

**Background:**

Switchgrass (*Panicum virgatum* L.) has a great potential as a platform for the production of biobased plastics, chemicals and energy mainly because of its high biomass yield on marginal land and low agricultural inputs. During the last decade, there has been increased interest in the genetic improvement of this crop through transgenic approaches. Since switchgrass, like most perennial grasses, is exclusively cross pollinating and poorly domesticated, preventing the dispersal of transgenic pollen into the environment is a critical requisite for the commercial deployment of this important biomass crop. In this study, the feasibility of controlling pollen-mediated gene flow in transgenic switchgrass using the large serine site-specific recombinase Bxb1 has been investigated.

**Results:**

A novel approach utilizing co-transformation of two separate vectors was used to test the functionality of the Bxb1/*att* recombination system in switchgrass. In addition, two promoters with high pollen-specific activity were identified and thoroughly characterized prior to their introduction into a test vector explicitly designed for both autoexcision and quantitative analyses of recombination events. Our strategy for developmentally programmed precise excision of the recombinase and marker genes in switchgrass pollen resulted in the generation of transgene-excised progeny. The autoexcision efficiencies were in the range of 22-42% depending on the transformation event and assay used.

**Conclusion:**

The results presented here mark an important milestone towards the establishment of a reliable biocontainment system for switchgrass which will facilitate the development of this crop as a biorefinery feedstock through advanced biotechnological approaches.

## Background

Biomass from perennial grasses is considered a “low-input high-diversity” feedstock [1] for the production of second generation biofuels in the US and Europe [2]. Switchgrass (*Panicum virgatum* L*.*), a warm-season perennial grass native to North America, is recognized as a premium candidate and has been identified by the U.S. Department of Energy as a model herbaceous energy crop due to its potential for high biomass production on marginal land, low agricultural inputs and positive environmental impacts [3]. Traditional breeding efforts have been focused on the improvement of switchgrass as a forage crop [4], while the current bioenergy research is targeting high cellulose and starch content for bioethanol production and low ash content for combustion systems [5] combined with significantly increased biomass yield. However, conventional breeding alone may not be able to meet the milestones for the commercial implementation of renewable biofuels as defined by the US government in the 2005 Energy Policy Act, the 2007 Energy Independence and Security Act and the 2008 Food, Conservation and Energy Act. Thus, a biotech approach to engineering bioenergy-related traits in switchgrass has been explored by many research groups [6]. Moreover, the recently demonstrated potential of switchgrass as a production platform for PHA bioplastics and biochemicals [6,7] makes this crop a promising candidate for the development of a value added biorefinery feedstock for the production of biobased coproducts and energy [8].

A major challenge for the commercialization of transgenic switchgrass and other outcrossing perennial grasses is to prevent the pollen-mediated transfer of transgenes to non-transgenic crops and wild relatives [9,10]. Although several biocontainment methods have been developed to reduce transgenic pollen dispersal, they have been tested mainly in model plants [11]. Among these methods, site-specific recombination is recognized as a promising strategy for transgene removal and other manipulations of the plant genome [12].

In this study, we have investigated the feasibility of engineering site-specific autoexcision of transgenes in switchgrass pollen mediated by the large serine recombinase Bxb1. Bxb1 is a 500 amino acid enzyme that binds minimal recognition sites 48 bp *attP* and 38 bp *attB* without the need for cofactors [13] to execute unidirectional site-specific recombination [14]. Essentially all reactions are fixed in place, which is advantageous for the stability of genomic engineering. The Bxb1/*att* system has previously been tested in tobacco [15], *Arabidopsis*[16] and wheat [17]. This is the first report demonstrating the functionality of a site-specific recombination system in switchgrass, an exclusively self-incompatible and highly heterozygous species.

## Results

### Bxb1/*att* recombination system is functional in switchgrass

**Figure 1:**
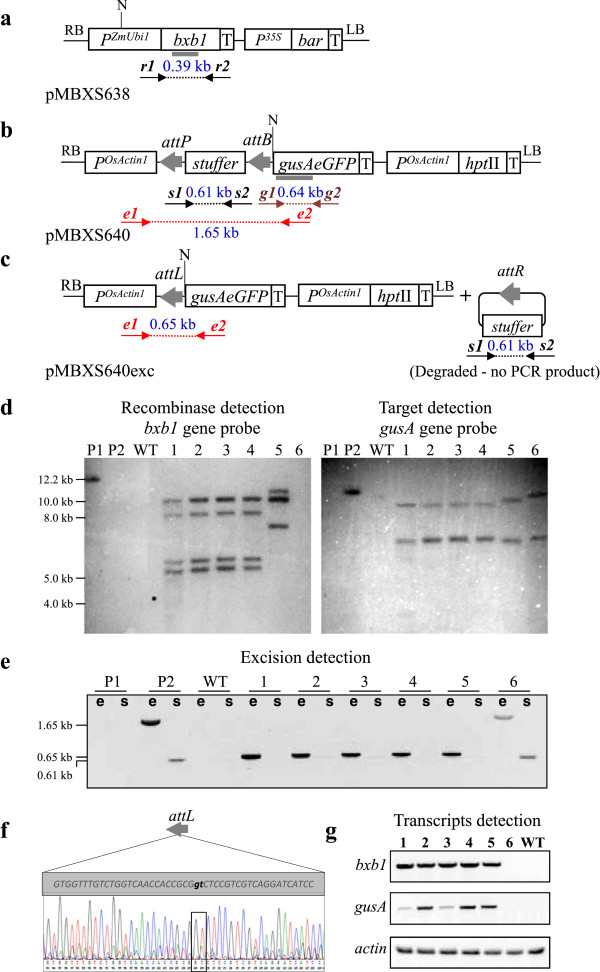
**Evaluation of Bxb1 functionality in switchgrass.** Schematic of **(a)** the pMBXS638 T-DNA containing the *bxb1* recombinase gene, **(b)** the pMBXS640 T-DNA carrying a non-coding DNA fragment (stuffer), and **(c)** the pMBXS640 predicted T-DNA structures after stuffer excision. PCR primers are shown as *r1&r2, g1&g2, e1&e2* and *s1&s2*, *att* sites as grey arrows and hybridization probes as grey rectangles. **(d)** Southern blot with probes for the *bxb1* gene (left panel) and the *gusA* gene (right panel). *Lanes*: *P1*, pMBXS638 DNA; *P2*, pMBXS640 DNA; *WT*, wild-type plant; *1*–*4*, individual co-transformed plants from the same callus line; *5*, co-transformed plant regenerated from another callus line; *6*, plant transformed with the vector pMBXS640. **(e)** PCR for detection of Bxb1-mediated excision of the stuffer: *e*, PCR reactions for the stuffer construct using primers annealing to sequences outside the region flanked by the *att* sites (primers *e1&e2*); *s*, PCR reactions for the stuffer fragment (primers *s1&s2*); *Lanes*: *P1*, pMBXS638 DNA; *P2*, pMBXS640 DNA; the plant samples are in the same order as in **(d)**. **(f)** Sequence of a PCR product (obtained with primers *e1&e2*) representing the *attL* footprint. **(g)** RT-PCR detection of *bxb1* (with primers *r1&r2* in 5 ng of total RNA), *gusA* (*g1&g2*, 200 ng RNA), and actin (*ActA* and *ActB*, 0.5 ng RNA) as a loading control. *Lanes*: *1*–*6*, transgenic plants in the same order as in **(d)** and **(e)**; *Abbreviations*: *P*^*ZmUbi1*^*,* promoter and first intron of the maize *ubiquitin1* gene; *bxb1*, Bxb1 recombinase gene; *P*^*35S*^, *CaMV*35S promoter; *bar*, marker gene conferring resistance to bialaphos; *P*^*OsActin*1^, promoter and first intron of the rice *actin1* gene; *gusAeGFP*, β-glucuronidase-enhanced Green Fluorescent Protein fusion; *attB* and *attP*, Bxb1 recombinase recognition sites; *attL* and *attR*, sequences formed after excision; *T*, transcription terminator; *LB*, left border sequence; *RB*, right border sequence; *N*, *Nco*I site.

A gain-of-function strategy was used to test the Bxb1/*att* site-specific recombination system in switchgrass. Two vectors, the recombinase vector pMBXS638 (Figure 1a) and the target vector pMBXS640 (Figure 1b), were created and used for co-transformation. Their design allows for the formation of a functional expression cassette of the β-glucuronidase-enhanced Green Fluorescent Protein (*gusAeGFP*) bifunctional reporter gene and *P*^*OsActin1*^ after Bxb1-mediated site specific recombination removes the stuffer DNA (Figure 1c).

The vectors were co-introduced into switchgrass callus cultures using a two-strain co-transformation method. Five co-transformants obtained from two independent callus lines were identified in tissue culture by PCR using primers specific for the coding regions of the transgenes. The co-transformed plants were transferred to soil and grown in a greenhouse for further analyses. Transgenic plants transformed with the vector pMBXS640 and wild-type plants (regenerated from untransformed callus cultures) grown under the same conditions were used as controls.Southern blot analysis confirmed that the plants obtained from different callus lines were independent co-transformation events. The results also revealed the presence of different copy numbers of the pMBXS638 and pMBXS640 T-DNAs (Figure 1d).

Bxb1-mediated excision of the stuffer in co-transformed plants was analyzed by PCR with the primers *s1&s2* specific for the stuffer sequence and *e1&e2* annealing to sequences outside the region flanked by the Bxb1 recombination sites (Figure 1c). This analysis revealed the lack of the stuffer in the co-transformed plants (Figure 1e). The size of the amplified fragment suggested precise excision of the DNA sequence between *attB* and *attP* and the formation of the predicted footprint *attL* was confirmed by sequencing of the PCR product (Figure 1f). RT-PCR analysis demonstrated the expression of the recombinase gene and the transcriptional activation of the reporter gene (Figure 1g). As expected, *gusA* and *bxb1* transcripts were not detected in the plant transformed with the stuffer vector pMBXS640 alone (lane 6 in Figure 1g).

### Characterization of the rice pollen-specific promoters *PS1* and *PS3* in switchgrass

In an effort to identify promoters suitable for transgene biocontainment in switchgrass, the spatiotemporal activity of two pollen-specific promoters from rice was evaluated in plants transformed with the vectors pGPro8-PS1 and pGPro8-PS3 which contain *PS1* and *PS3* promoter-GUSPlus reporter gene fusions, respectively.

**Figure 2:**
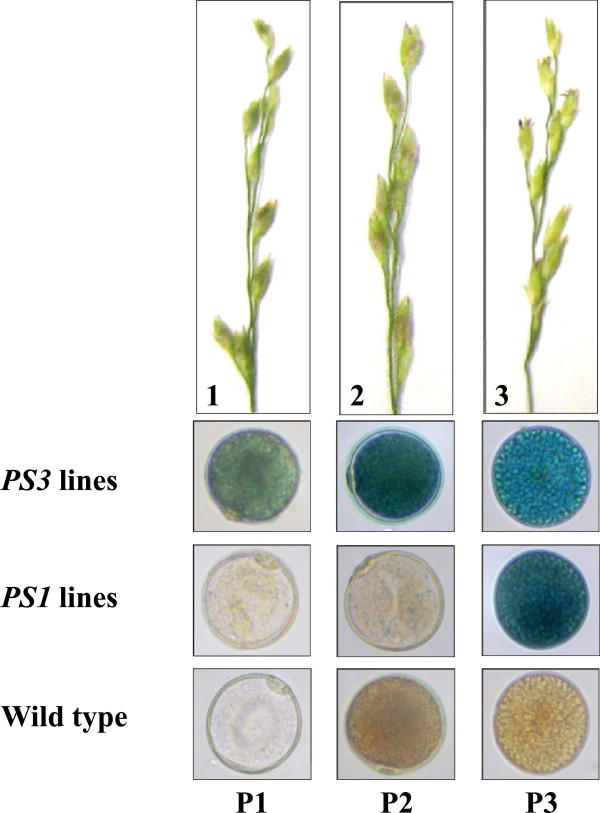
**Activity of the rice pollen-specific promoters *PS1 *and *PS3 *in switchgrass pollen.***1*–*3*, Images of switchgrass spikelets showing their morphology at representative developmental stages before anthesis (*1*–*2*) and at anthesis (*3*) corresponding to the stages of pollen development (P1 and P2) and maturation (P3) according to [18]. The stained pollen grains were examined at magnification of 63×.

To test the activity of these promoters in vegetative tissues, 27 plants representing 6 pGPro8-PS1 events and 36 plants representing 12 independent pGPro8-PS3 events were analyzed in tissue culture by histochemical staining for GUS activity and by RT-PCR. No expression of the reporter gene was detected in leaves and roots (data not shown). Randomly selected transgenic plants from 6 independent transformation events for each promoter construct were grown in soil. At the reproductive stage, reporter gene expression was monitored in the perfect terminal and vestigial lower florets of spikelets before, at, and after anthesis by histochemical staining. GUS activity was observed at different stages of pollen development – uninuclear microspores and bicellular pollen grains as well as in mature tricellular pollen grains in the *PS3* transgenic lines, while strong expression of the reporter gene driven by the rice *PS1* promoter was detected only in mature pollen (Figure 2).

### Bxb1-mediated transgene autoexcision in switchgrass pollen

**Figure 3:**
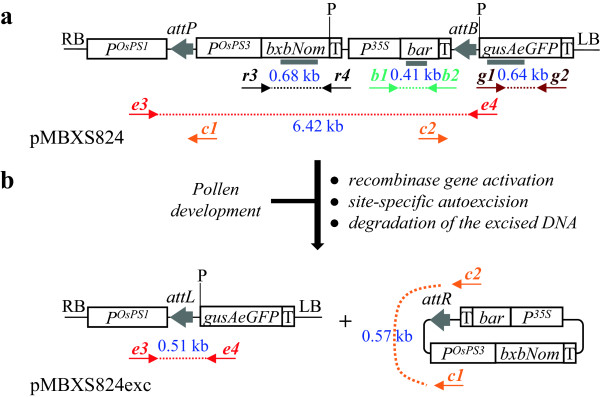
**Schematic illustration of Bxb1 recombinase-mediated transgene autoexcision in pollen. (a)** T-DNA of the vector pMBXS824. PCR primers are indicated as *r3&r4*, *b1&b2*, *g1&g2*, *e3&e4*, and *c1&c2* (Table 3), *att* sites as grey arrows and hybridization probes as grey rectangles. **(b)** Predicted pollen-specific autoexcision event and T-DNA fragments. *P*^*OsPS3*^ drives the *bxbNom* gene expression during pollen development and *P*^*OsPS1*^ drives the *gusAeGFP* gene expression after autoexcision. *Abbreviations*: *P*^*OsPS1*^ and *P*^*OsPS3*^, pollen specific promoters; *bxbNom*, optimized Bxb1 recombinase gene; *P*, *Pst*I restriction site; other abbreviations are the same as in Figure 1.

A gain-of-function strategy based on pollen-specific autoexcision was also used to obtain switchgrass plants with transgene-excised pollen. The test vector pMBXS824, harboring the expression cassettes of the optimized recombinase gene *bxbNom* and the marker gene *bar* was created for this purpose (Figure 3a). To facilitate the identification of autoexcision events, the two expression cassettes, flanked by the Bxb1 recombination sites, separate the *PS1* promoter (proximal to *attP50* site) from the reporter gene *gusAeGFP*, which is silent prior to the removal of the transgenes between the *att* sites. The events leading to the pollen-specific elimination of the recombinase and marker genes from the plant genome (autoexcision) are illustrated in Figure 3b.

Switchgrass transformation with pMBXS824 yielded 26 bialaphos-resistant callus lines and 78 primary transformants regenerated from them were identified by PCR. In total, 38 transgenic plants from 22 independent transformation events were grown in a greenhouse and analyzed by molecular and histochemical techniques to identify plants with Bxb1-mediated autoexcision in pollen. For this purpose, pollen grains were collected from perfect florets at anthesis and used for DNA and RNA isolation as well as for GUS activity staining.

**Figure 4:**
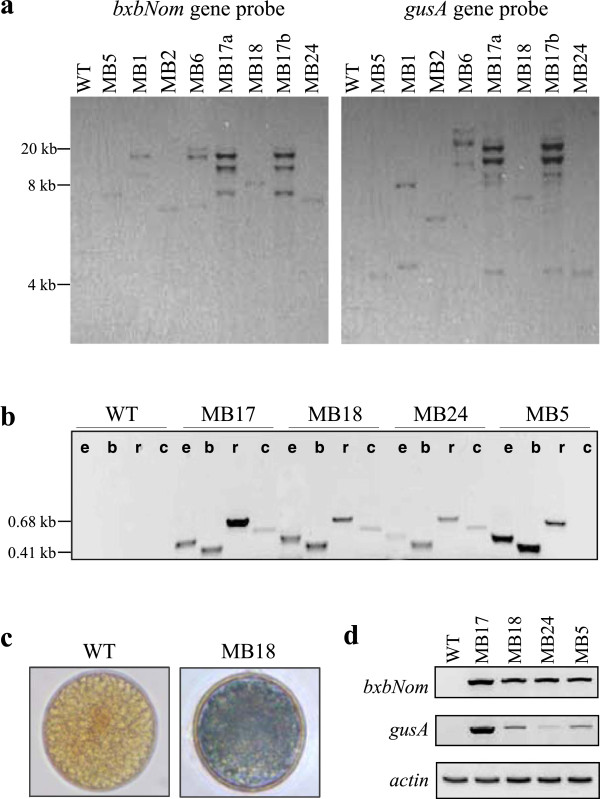
**Analyses of T**_**0 **_**plants with transgene autoexcision in pollen. (a)** Determination of the copy number of the pMBXS824 T-DNA (Figure 3**a**) by Southern blot hybridization of genomic DNA isolated from leaves of transgenic and control plants. *Lanes*: *WT*, a non-transgenic plant; *MB*, individual transgenic plants from lines MB1, 2, 5, 6, 17a, 17b, 18, and 24. **(b)** PCR analysis for detection of Bxb1-mediated autoexcision in pollen from select transgenic lines. Genomic DNA was isolated from mature pollen grains collected from perfect florets at anthesis. *PCR reactions*: designated lane *e*, detection of autoexcision (primers *e3&e4*, PCR product size 0.51 kb); *b*, detection of the marker gene *bar* (primers *b1&b2*, 0.41 kb); *r*, detection of the recombinase gene *bxbNom* (primers *r3&r4*, 0.68 kb); *c*, detection of the excised DNA fragment (primers *c1&c2*, 0.57 kb). **(c)** Detection of GUS activity in mature pollen (magnification 63×). **(d)** RT-PCR for detection of the *bxbNom* and *gusA* transcripts in pollen. The following amounts of total RNA per reaction were used: 10 ng for the *bxbNom* gene, 25 ng for the *gusA* gene, and 2 ng for actin as a loading control. The plant samples are in the same order as in **(b)**.

Seven switchgrass lines with transgene excision in pollen were identified by PCR using primers *e3&e4* (Figure 3). A single copy of the inserted T-DNA was detected in four of these lines by Southern blot hybridization (Figure 4a). A more detailed PCR analysis was conducted with four transgenic lines using different sets of primers (Figure 3) for detection of autoexcision, *bxbNom*, *bar*, and the excised DNA fragment. The results revealed the presence of the predicted 0.51 kb band obtained after excision along with the recombinase and marker genes (Figure 4b). In all lines, the excision PCR product contained the expected *attL* footprint as verified by sequencing (data not shown). Since no strong GFP fluorescence was detected in switchgrass pollen, all reporter gene analyses consisted of evaluating transgenic plants for *gusA* gene expression. Although GUS activity was detected in some mature pollen grains (Figure 4c), the observed staining patterns were not conclusive. A possible explanation could be reduced expression of the reporter gene due to the presence of the *attL* sequence between the promoter and the coding region in the newly-formed expression cassette as suggested previously [19]. Pollen-specific autoexcision resulting in expression of the *gusA* gene was demonstrated by RT-PCR with total RNA isolated from pollen (Figure 4d). The observed accumulation of the *bxbNom* transcripts confirmed the presence of the recombinase gene detected by PCR.

### T_1_ progeny analyses

Since switchgrass is an exclusively cross-pollinating species, T_1_ seeds were obtained from controlled crosses between transgenic plants (MB5, MB17, MB18 and MB24) and wild type plants representing different Alamo genotypes. In these reciprocal crosses, the T_0_ plants were used as both male (WT × MB) and female (MB × WT) parents.

In one set of experiments, PCR was used to distinguish between transgenic and non-transgenic T_1_ plants. Since the Bxb1-mediated autoexcision occurs in the male gametes, transgene-excised progeny are only expected to be obtained from crosses with T_0_ plants used as the pollen donor. In the offspring from the reciprocal crosses (MB × WT), the transgenes are expected to be transmitted through maternal inheritance and to be present in the genome of the transgenic T_1_ plants.

**Figure 5:**
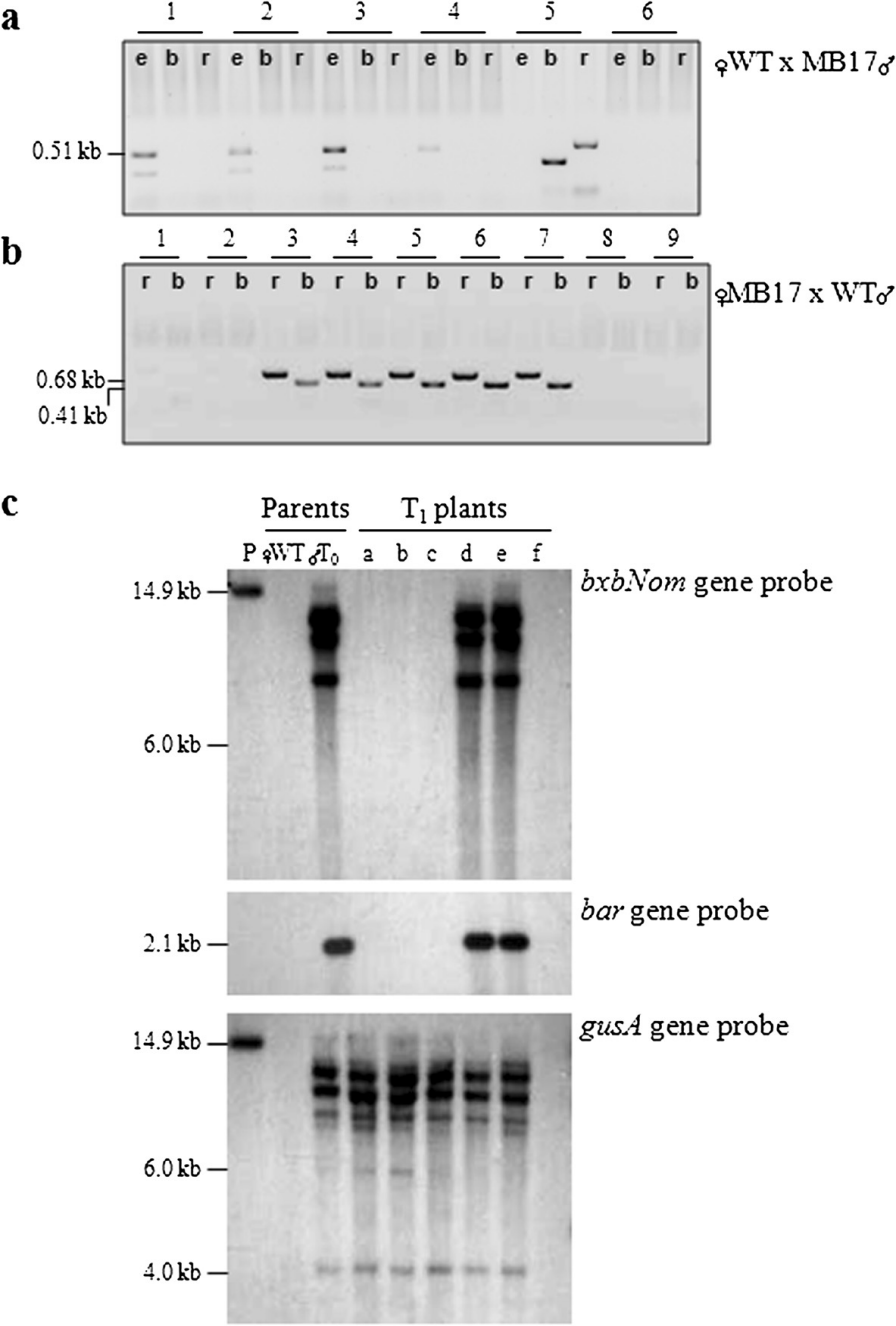
**Molecular analyses of T**_**1 **_**plants from controlled crosses between MB17 and non-transgenic plants. (a)** PCR for identification of transgene-excised progeny when MB17 was used as the male parent (WT × MB17 cross). *1*–*6*, individual T_1_ plants. *Lanes*: *e*, PCR reactions for detection of autoexcision (primers *e3&e4*); *b*, reactions for the marker gene *bar* (primers *b1&b2*); *r*, reactions for the recombinase gene *bxbNom* (primers *r3&r4*). **(b)** PCR for determination of the segregation ratio in the T_1_ generation when MB17 was used as the female parent (MB17 × WT cross). *1*–*9*, individual T_1_ plants. *PCR reactions*: same as reactions *r* and *b* in **(a)**. **(c)** Southern blot hybridization for detection of the pMBXS824 T-DNA (Figure 3) in the genome of WT × MB17 progeny. *Lanes*: *P,* pMBXS824 DNA; *WT*, wild-type plant used as the female parent; *T*_*0*_, MB17 plant used as the pollen donor; *a-f*, individual T_1_ plants. Based on the results from PCR screening (Table 1), the analyzed T_1_ plants were categorized as follows: *a-c*, transgene-excised; *d-e*, transgenic; *f*, a non-transgenic T_1_ plant (a null segregant).

In total, 556 T_1_ plants obtained from crosses of three lines with pollen-specific autoexcision were analyzed by PCR. The results from the molecular analyses of MB17 progeny are shown in Figure 5. Fifteen transgene-excised WT × MB17 seedlings were identified based on the presence of the 0.51 kb excision positive amplicon (see Figure 3) combined with the lack of the *bxbNom* and *bar* genes (some shown in Figure 5a). The offspring from the reciprocal crosses (87 T_1_ plants) were also screened to confirm the segregation ratio (Figure 5b). The complete elimination of the *att*-flanked target DNA from the genome of transgene-excised progeny was confirmed by DNA blot hybridization. As shown in Figure 5c for randomly selected WT × MB17 progeny, the recombinase and marker genes were not detected in T_1_ plants identified as transgene-excised by PCR. The reporter gene located on the pMBXS824 T-DNA but not included in the *att*-flanked fragment was detected in the transgenic progeny with the *gusA* gene probe (Figure 5c).

For all lines, the obtained segregation ratios of transgenic-to-non-transgenic T_1_ plants were analyzed by *χ*^2^-test (Table 1). Complete excision of the marker and recombinase genes was detected via genomic PCR in 23 of the transgenic WT × MB18 plants resulting in an autoexcision efficiency of 23.5% (23 of 98). No transgene elimination was observed in the progeny of MB5 possibly due to the insufficient number of T_1_ plants analyzed. The approximate 3:1 segregation ratio in the offspring of the multi-copy line MB17 when used as the male parent indicated that the transgenes were integrated in two loci as confirmed by the analysis of the progeny from the reciprocal cross. Fifteen transgene-excised plants representing 22.4% of the transgenic WT × MB17 T_1_ plants were identified (Table 1).

**Table 1:** **Segregation analysis of the T**_**1 **_**progeny from reciprocal controlled crosses**

	**Number of T**_**1 **_**plants**					
**Cross**	**Total**	**Transgenic (TR)**	**Transgene-excised**	**Non-transgenic (NT)**	**Ratio TR:NT**	***χ***^**2**^
WT × MB18	183	98	23	85	1:0.9	0.92
MB18 × WT	112	61	NA	51	1:0.8	0.89
WT × MB5	42	22	0	20	1:0.9	0.10
MB5 × WT	33	16	NA	17	1:1.1	0.03
WT × MB17	99	67	15	32	3:1.4	2.83
MB17 × WT	85	58	NA	27	3:1.4	2.07

The T_1_ plants were analyzed by genomic PCR using primers *e3&e4*, *b1&b2*, *r3&r4*, and *g1&g2* (see Table 3). *MB*, switchgrass plants engineered for pollen-specific transgene autoexcision; *NA* not applicable, *WT* wild-type plants.

For functional analyses of autoexcision events, 344 T_1_ seedlings obtained from reciprocal controlled crosses of two transgenic lines were treated with the herbicide Basta (Table 2). Since both non-transgenic and transgene-excised plants would be Basta sensitive, the excision efficiency was estimated based on the expected segregation ratio. In the offspring of the single-copy line MB24, 50% of the plants (38 of 76) should be non-transgenic and 50% - transgenic (1:1 segregation ratio). In WT × MB24 progeny, 25 plants were tolerant to the herbicide due to expression of the *bar* gene. Assuming that 38 of the 51 Basta sensitive plants were non-transgenic, 13 plants were presumed to be transgene-excised. They represented 34.2% of the expected number of transgenic plants. The autoexcision efficiency of 41.9% in the offspring of the multi-copy line MB17 (when used as the pollen donor) was calculated considering the ratio of 3:1.4 obtained by PCR analysis (Table 1, Table 2).

**Table 2:** **Functional analyses of autoexcision events in the T**_**1 **_**generation**

	**T**_**1 **_**plants assayed for Basta tolerance**			**Transgene-excised T**_**1 **_**plants**	**Estimated autoexcision efficiency [%]**
**Cross**	**Total**	**Tolerant**	**Sensitive**		
WT × MB24	76	25	51	13	34.2
MB24 × WT	91	45	46	NA	NA
WT × MB17	126	50	76	36	41.9
MB17 × WT	51	31	20	NA	NA

The number of transgene excised plants was estimated based on the segregation ratios. *NA* not applicable, *NT* non-transgenic plants, *TR* transgenic plants.

## Discussion

Plant genetic engineering provides a powerful tool for crop improvement and the development of crop-based production systems. However, the potential dispersal of the introduced transgenes via different biological vectors (pollen, seeds, and/or vegetative propagules) is a key issue for the commercial use of transgenic crops. Since most forage and turf grasses, including many promising bioenergy crops, are exclusively outcrossing and less domesticated, the risk of transgene transfer from these species to their wild relatives is very high, which raises commercial and environmental concerns [9,10,20,21]. Although different biological containment strategies have been proposed and developed, most of them only have a proof of concept in model plants. Some of these strategies utilize site specific recombination systems for the removal of superfluous DNA from the genome of genetically engineered plants [12,22,23].

In this study, we have demonstrated the possibility to reduce pollen-mediated transgene flow in switchgrass using developmentally programmed site-specific recombination for transgene autoexcision from the plant genome. In general, this strategy requires a combination of pollen- or microspore-specific promoters and highly active recombinases suitable for the targeted crop. Due to the lack of such information for switchgrass, our research started with the identification and functional characterization of potential candidates. Two recombination systems - Bxb1/*att* and ParA/*MRS*[12] - and a set of four promoters active in monocot floral and reproductive organs – the barley *Lem1* promoter [24,25] and the rice promoters *LP2*, *PS1*[26,27], and *PS3* - have been tested in stably transformed switchgrass plants in tissue culture and soil. Based on this initial screening (data not shown), we have chosen the well characterized large serine recombinase Bxb1 [14,28] with demonstrated activity in different plant species [15-17], and the rice pollen-specific promoters *PS1* and *PS3*.

A novel approach was used to test the Bxb1*/att* recombination system in switchgrass. A recombinase activity detection system based on two transformation vectors was designed for this purpose. The target and recombinase vectors were co-introduced into callus cultures resulting in the production of stably co-transformed plants with precise (without the gain or loss of nucleotides) excision of the *att*-flanked DNA fragment (Figure 1). When the recombinase gene and the target DNA (a marker and/or a gene of interest) are located on different vectors, they can be combined in a transgenic plant by re-transformation or genetic crosses or transient expression of the recombinase gene [29]. Our results demonstrate the possibility of achieving the complete removal of undesired DNA from the switchgrass genome in the same time frame as required for our standard transformation protocol. Moreover, no crosses between transgenic lines were necessary, which is advantageous for genetic engineering of switchgrass and other self-incompatible crops with a long reproductive cycle and overall complex genetics due to high levels of ploidy and heterozygosity. To our knowledge, this is the first report on engineering site specific recombinase-mediated excision in a monocot species by co-transformation.

After successfully validating the functionality of the Bxb1/*att* recombination system in switchgrass, we evaluated the spatiotemporal activities of candidate promoters prior to their use for pollen-specific autoexcision. The rice *PS1* and *PS3* promoters did not show any background activity in somatic tissues detectable by molecular and histochemical techniques. The observed differences in the functionality profile of these promoters in switchgrass pollen (Figure 2) led us to choose the *PS3* promoter for achieving high expression of the Bxb1 recombinase during pollen development and maturation.

To evaluate whether transgene flow via pollen in switchgrass can effectively be controlled by Bxb1-mediated site specific recombination, the autoexcision test vector pMBXS824 was created with the expression cassettes of the recombinase and marker genes positioned between the *att* target sequences (Figure 3). For more effective performance, an optimized form of the *bxb1* coding sequence (*bxbNom*) was used that contained a C-terminal nuclear localization signal [17]. The developmentally programmed pollen-specific *bxbNom* expression was engineered by placing the *PS3* promoter upstream of the recombinase gene. For screening purposes, pMBXS824 also contains the rice pollen-specific promoter *PS1* and the reporter *gusAeGFP* gene outside of the *att*-flanked target DNA fragment. Activation of *bxbNom* expression by the *PS3* promoter during pollen development led to Bxb1-mediated autoexcision of the genetic material between the *att* recombination sites. As a result, a new expression cassette is formed where the *PS1* promoter and the *attL* footprint are fused to *gusAeGFP* reporter gene within the heritable pollen genome.

In total, 22 independent transgenic switchgrass lines were used to examine the efficiency of the autoexcision strategy. Transgene-excised pollen was detected in 7 of these lines using PCR and GUS activity staining. The results from DNA blot analysis demonstrated that four of these T_0_ lines contained a single copy of pMBXS824 T-DNA, while the other lines had 2–5 copies (Figure 4a). Three of the single-copy lines (MB5, MB18 and MB24) and the multi-copy line MB17 were used for more detailed molecular and genetic analyses. PCR analysis of genomic DNA isolated from mature pollen grains revealed the presence of the *bxbNom* and *bar* genes in all of the transgenic lines along with the 0.51 kb amplicon that indicates site-specific autoexcision. The minicircle excision PCR product (Figure 3b) was detected in three of the lines, MB17, MB18 and MB24 (PCR reactions *c* in Figure 4b). These circular DNA molecules formed upon site-specific recombination are expected to be lost via degradation and cell division [30] but they may be stable in non-proliferating cells, such as mature pollen grains. The excised DNA fragments and/or the genomic DNA of pollen without autoexcision could serve as template for the amplification of the *bar* and *bxbNom* transgenes observed in our experiments. The presence of the recombinase gene in the pollen genome was also confirmed by the detection of its transcripts by RT-PCR (Figure 4d). Taken together, the results suggest that either complete autoexcision has not occurred in all of the transgenic pollen grains, or that there is retention of the excised DNA. An optimized recombinase-promoter combination designed specifically for switchgrass could ensure complete removal of the transgenes in the germline or microspores (see Conclusions), while the minicircles would be degraded in the actively proliferating cells of the developing embryo and growing seedling.

The efficiency of Bxb1-mediated transgene autoexcision in switchgrass pollen or transmission of the resulting recombination event to the next generation was assessed by molecular and functional analyses. T_1_ progeny was obtained from reciprocal crosses between transgenic lines and wild-type plants from different genotypes to mimic cross pollination in natural switchgrass populations [7]. To date, pollen-specific autoexcision systems have only been demonstrated in diploid self-pollinating model plants (see below), and only the offspring of single-copy events have been analyzed in detail. Since all transgenic and wild-type switchgrass plants used in our study are from the tetraploid cultivar Alamo, we have also included a multi-copy line along with the single-copy ones to illustrate the complexity of progeny analyses and the potential of Bxb1-mediated autoexcision in a polyploid heterozygous crop. The MB17 line contains at least 5 copies of the pMBXS824 T-DNA and some of them are probably arranged as tandem repeats based on the DNA blot hybridization analysis (Figure 4a) and the transgene segregation observed in the T_1_ generation. The progeny segregation ratio (Table 1) indicates that the T-DNAs are integrated in two unlinked loci, which can potentially result in the deletion or rearrangement of genomic DNA fragments between different transgenic loci upon Bxb1-mediated recombination in the pollen. Based on morphological observations, if genomic DNA has been removed or rearranged, it has not caused detectable changes in the phenotype and development of the analyzed WT × MB17 progeny. On the other hand, the unaffected alleles present in the polyploid switchgrass genome might compensate for any DNA loss.

Overall, the results from progeny analyses revealed that the offspring of all lines segregated in the expected Mendelian ratios for transgenic:non-transgenic plants suggesting that the Bxb1-mediated site specific recombination did not affect pollen viability and germination. The identification of transgene-excised and non-excised transgenic T_1_ plants in the offspring of each line (Table 1) is in agreement with the detected presence of both autoexcision products and transgenes in T_0_ pollen. Consistent with the transmission of the recombined allele(s) through the male gametes, transgene-excised progeny were only obtained from controlled crosses when the primary transformants were used as pollen donors. The identified transgene-excised T_1_ plants represented approximately 22-42% of the offspring depending on the transformation event and the assay used. Interestingly, similar autoexcision efficiencies were observed in the progeny of single- and multi-copy lines (Tables 1 and 2).

Pollen-specific autoexcision strategies have previously been reported in tobacco and *Arabidopsis*[21,29,31,32]. Germline-, microspore- and pollen-specific promoters have been utilized for developmentally regulated expression of CRE/*loxP*, CinH/*RS2* or FLP/*FRT* recombination systems resulting in high frequency (up to ~100%) of production of transgene-excised plants in the progeny (T_1_-T_3_ generations) obtained through selfing or backcrossing of primary transformants. The applicability of these approaches in other plant species and crops, however, is yet to be evaluated.

## Conclusions

This is the first study demonstrating a functional and relatively simple strategy for the reduction of pollen-mediated gene flow in transgenic switchgrass. Our results indicate that the Bxb1/*att* site specific recombination system can be used for the precise removal of the recombinase and marker genes from switchgrass pollen resulting in the generation of transgene-excised progeny. The genetic elements included in our test vector for screening purposes can be eliminated from an optimized autoexcision vector to obtain plants with transgene-free pollen. Although the Bxb1 recombinase has been shown to have activity in other plant species, no Bxb1-mediated autoexcision has previously been reported. In addition, we also provide experimental evidence that our Bxb1-based autoexcision vector can be used for the transcriptional activation of a gene of interest at a defined developmental stage. A similar strategy utilizing an *attP* and *attB* flanked selectable marker combined with tissue-specific Bxb1 expression could be used to generate marker-free transgenic switchgrass that contain only the introduced genes of interest.

Further studies are necessary to improve the recombination efficiency in switchgrass pollen. Consistent with results from research with model plants, the results presented here clearly indicate that high levels of recombinase activity conferred by promoters with crop-specific functionality is crucial for the successful elimination of transgenes in gametes or somatic tissues. Our future work on the improvement of the Bxb1/*att* system will include codon optimization of the recombinase gene sequence for increased expression specifically in switchgrass. Intron-mediated enhancement of the *bxb1* gene expression [33] is also considered. Another approach to improving the Bxb1 activity would be mutagenesis by a combination of error prone PCR and alanine scanning which could both increase the recombination rate and/or modify the native binding preferences of the enzyme as reported previously for the related recombinase phiC31 [34]. The outcome of these strategies would also depend on the activity and tissue specificity of the promoter controlling the recombinase gene expression. As described above, the highest autoexcision efficiencies have been achieved in dicotyledonous plants using promoters specifically active in germline or during microsporogenesis. However, monocot promoters with this type of spatiotemporal activity are not currently available, which combined with the lack of thorough analyses of major aspects of switchgrass gene flow hinder the development of both risk assessment strategies and versatile gene biocontainment systems for this valuable bioenergy crop.

## Methods

### Construction of plant transformation vectors

All gene constructs were made using standard molecular biology techniques. Five binary vectors were used for switchgrass transformation and co-transformation.

The vectors pMBXS638 and pMBXS640 were co-introduced in callus cultures to test the functionality of the Bxb1/*att* recombination system in switchgrass. The vector pMBXS638 contains the *bxb1* recombinase gene driven by *P*^*ZmUbi1*^ (the promoter and first intron of the maize *ubiquitin1* gene) and the *bar* gene (conferring resistance to bialaphos) as a marker for plant selection. The target vector pMBXS640 was constructed using the vector pGPro1 [35] containing the *hptII* gene (conferring resistance to hygromycin) as a plant selectable maker. The vector also harbors a 0.69 kb non-coding DNA fragment (stuffer) flanked by the Bxb1 recombination sites *attP50* and *attB42* in direct orientation, *P*^*OsActin1*^ (the promoter and first intron of the rice *actin1* gene) proximal to *attP50*, and a distal β-glucuronidase-enhanced Green Fluorescent Protein (*gusAeGFP*) bifunctional reporter gene [35]. For complete sequences of the Bxb1 attachment sites (*att*), see [28].

The vectors pGPro8-PS3 and pGPro8-PS1 were used to evaluate the spatiotemporal activity patterns of the rice pollen specific promoters *PS3* and *PS1,* respectively. A 2.2 kb fragment representing the *PS3* promoter sequence [EMBL: JN593331] was fused to the reporter β-glucuronidase gene *GUSPlus*[36] to assemble the vector pGPro8-PS3. The vector pGPro8-PS1 harbors the 1.8 kb sequence of the *PS1* promoter [GenBank: JN593329] [26,27] driving the *GUSPlus* gene. Both vectors contain the *hptII* gene as a marker for callus and plant selection under the control of the promoter and first intron of the rice *ubiquitin2* gene (*P*^*OsUbi2*^) [7,37]. The presence of the transgenes in hygromycin-resistant putative transformants was confirmed by PCR using primers *g3&g4* and *h3&h4* (Table 3).

**Table 3:** Primers used in this study

**Primer ID**	**Gene/element**	**Sequence (5′- 3′)**	**Reference**
*b1*	*bar*	GCACCATCGTCAACCACTACATCG	[7]
*b2*		TCATGCCAGTTCCCGTGCTTG	[7]
*h1*	*hptII (pMBXS640)*	TGATCGAAAAGTTCGACAGCGTCTC	This study
*h2*		CTCCAGTCAATGACCGCTGTTATGC	This study
*h3*	*hptII (pGPro8)*	CTCCCGATTCCGGAAGTGCT	This study
*h4*		CATCGCCTCGCTCCAGTCAA	This study
*r1*	*bxb1*	CTGGTAGTCATCCGCCTGT	This study
*r2*		CTTCTAATTCCATCTGCGCCAC	This study
*r3*	*bxbNom*	CAACTGGAGAGCTGCCAACAAC	This study
*r4*		ATATCCCAGCATGGCTTCGG	This study
*g1*	*gusAeGFP*	AGCGCGTTACAAGAAAGC	This study
*g2*		AGAGATAACCTTCACCCGG	This study
*g3*	*GUSPlus*	CGCAACCATATCGGATATGTCT	This study
*g4*		TGACATTCGGAATCTCCACG	This study
*s1*	Stuffer	CTCGATCGGTTAGCATAGGCAG	This study
*s2*		GACTTAGCGTAGCAATGGCAACTG	This study
*e1*	Stuffer construct	TGTAGTCTAGAGTGCTCCATTC	This study
*e2*		CGGCTTCAAATGGCGTATAGC	This study
*e3*	Autoexcision	TCTAGAGTGCTCCATTCTCTCTCCTCG	This study
*e4*		GTTAAAACTGCCTGGCACAGCAATT	This study
*c1*	Excised DNA fragment	GGGTTCCTATAGGGTTTCGCTCATG	This study
*c2*		TGATTTACCAGAAGCGGAGGAAGAA	This study
*ActA*	*actin*	CACTGGAATGGTCAAGGATG	[7]
*ActB*		CTCCATGTCATCCCAGTTG	[7]

The vector pMBXS824 was constructed for Bxb1-mediated transgene autoexcision in switchgrass pollen and identification of transgene-excised progeny. The vector harbors the expression cassettes of an optimized *bxb1* coding sequence (*bxbNom*) [17] driven by the *PS3* promoter and the marker gene *bar* under the control of the *CaMV 35S* promoter between the Bxb1 recombination sequences. The *PS1* promoter and the reporter gene *gusAeGFP* are positioned outside of the *att*-flanked DNA fragment.

### Plant material, transformation and co-transformation

Switchgrass plants from Alamo genotype 56 [7] grown under greenhouse conditions were used for initiation of immature inflorescence-derived callus cultures. The top culm nodes of elongating tillers with 3–4 visible nodes were used for development of inflorescences in tissue culture following a previously published procedure [38]. Callus cultures were initiated from individual spikelets from *in vitro* developed panicles and propagated by transferring on to a fresh medium for callus growth every four weeks. These highly embryogenic immature inflorescence-derived callus cultures were transformed with *Agrobacterium tumefaciens,* strain AGL1 and selected with 10 mg l^-1^ bialaphos following the previously published protocols [7,39,40].

For *Agrobacterium*-mediated co-introduction of transgenes on two independent T-DNAs located on two binary vectors in different bacterial cells, callus cultures were incubated in a mixture of equal volumes of *A. tumefaciens* (strain AGL1) cultures (OD_600_ 0.4) under the conditions described previously [7,39,40]. Co-transformed callus cultures and plants were subjected to double selection with 10 mg l^-1^ bialaphos and 200 mg l^-1^ hygromycin for 2–4 months with transfers to a fresh selection medium every two weeks.

The growth conditions in tissue culture and soil were as described previously [7,40]. Wild-type plants obtained from untransformed callus cultures and grown under the same conditions were used as controls.

### Molecular analyses

Primary switchgrass transformants and co-transformants were identified by PCR with total nucleic acid extracts from leaves of plants in tissue culture (REDExtract Plant PCR Kit, Sigma-Aldrich, St. Louis, MO, USA) using primers specific for the coding regions of the transgenes (Table 3) and the amplification conditions described previously [7]. The same procedure was used for PCR screening of T_1_ plants. Excision and autoexcision were detected by PCR with genomic DNA isolated from leaves and pollen, respectively, of soil-grown plants using a Wizard Genomic DNA Extraction kit (Promega, Madison, WI, USA) according to the manufacturer’s instructions. Primer pairs are specified in the text.

Southern blot hybridizations were performed with 15 μg of genomic DNA digested with *Nco*I for analysis of co-transformed plants and with *Pst*I for analysis of primary transformants and T_1_ plants following a previously described procedure [7,40].

### Expression analysis

For detection of the reporter and recombinase gene transcripts by reverse transcription-polymerase chain reaction (RT-PCR), total RNA was isolated from leaf tissues or pollen grains using the RNeasy Plant Mini Kit (Qiagen, Valencia, CA, USA) according to the manufacturer’s instructions. After DNase treatment and column purification, different amounts of RNA (specified in figure legends) were subjected to reverse transcription and DNA amplification in a one-step RT-PCR assay (Qiagen, Valencia, CA, USA) as described previously [7] using the primers listed in Table 3.

### Histochemical GUS staining

For detection of β-glucuronidase (GUS) activity, transverse leaf and root sections from plants in tissue culture or pollen grains from soil-grown plants were incubated in X-Gluc (5-bromo-4-chloro-3-indolyl-β-D-glucuronic acid; Gold Biotechnology, Inc., St. Louis, MO, USA) solution [41] at 37°C for 24 or 48 hours.

### Progeny analyses

T_1_ seeds were obtained from controlled crosses between transgenic and wild-type plants as reported previously [7,40] and germinated after stratification at 4°C for two weeks. The resulting seedlings were transferred to soil and analyzed by PCR and Southern blot hybridization as described above. Preliminary PCR experiments showed no amplification of the excision product in T_1_ plants obtained from crosses when the primary transformants with transgene-excision in pollen were used as the female parents.

T_1_ seedlings were treated with the herbicide Basta as described previously [7,40].

The segregation ratios were analyzed by the *χ*^2^-test at P = 0.05.

## Competing interests

The authors declare that they have no competing interests.

## Authors’ contributions

MNS designed the study, performed molecular analyses, analyzed data and drafted the manuscript. CAX carried on the design and construction of the autoexcision vector, switchgrass transformation and co-transformation, genetic and molecular analyses, characterized monocot promoters, analyzed data and participated in manuscript editing. KPR performed expression analyses, participated in construction of vectors and manuscript editing. RT identified, cloned and characterized the pollen-specific promoters, built the promoter-reporter gene pGPro8 binary vector constructs and edited drafts of the manuscript. OP and KDS contributed to the overall research concept of the project and edited drafts of the manuscript. JT participated in manuscript drafting, editing, study design, optimizing and testing of the recombinases. All authors read and approved the final version of this manuscript.
